# Developing comprehensive course evaluation guidelines in Tehran University of Medical Sciences

**Published:** 2015-07

**Authors:** ROGHAYEH GANDOMKAR, AZIM MIRZAZADEH, MOHAMMAD JALILI, LEYLA SADIGHPOUR

**Affiliations:** 1 Medical Education Department, Medical School, Tehran University of Medical Sciences, Tehran, Iran;; 2 Prosthodontics Department, Dentistry School, Tehran University of Medical Sciences, Tehran, Iran

## Dear Editor

Program directors need to evaluate educational programs to ensure their quality (1). The results of a survey conducted in Tehran University of Medical Sciences (TUMS) in early 2012 showed that program evaluation was not an established process in the majority of schools. It was at best a stand-alone project focusing on a single course or a particular component of it with no structured follow-up (2). Hence, we decided to promote and organize course evaluation practices in our university by development of general guidelines.

TUMS has eleven affiliated schools of various sizes and scope of activities. It was important to propose guidelines that were general enough to provide acceptable degree of consistency and coherence among evaluation activities in schools, and yet specific enough to enable schools to have their own evaluation plan tailored to their needs. In this regard, the project taskforce decided to consider the related literature on the existing program evaluation standards and guidelines as the starting point and formulate guidelines aligned with TUMS condition.

After generating the draft for the guidelines by taskforce, it was distributed among decision makers in all schools and their comments were obtained. Once the guideline was revised based on the suggestions, it was approved by the university Education Council in November 2012. In total, 22 guidelines categorized in 3 domains including course evaluation “infrastructures”, “design and implementation”, and “reporting and utilization of the results” were developed (2).

After sending the guidelines to schools, a comprehensive program evaluation workshop was conducted for the schools’ delegates. Afterwards, each school designed its own course evaluation plan based on the university guidelines. Course evaluation plans were appraised by taskforce and, if necessary, feedback was provided through a formal letter, face to face meeting or telephone conversation. 

Development of guidelines was a valuable approach to reach a common understanding of course evaluation between stakeholders in our university. There is usually an inadequate understanding of what course evaluation is and the concept is frequently reduced to teacher evaluation or student assessment (3). In spite of creating consistency of evaluation activities in our institution, the guidelines were not prescriptive and the schools were allowed to design their own plans adapted to their context, which is vital for a large institution such as TUMS with diverse cultural contexts.

We believe our approach has made changes in individuals’ thinking as well as the culture of schools involved in the process of development of course evaluation guidelines. The next step is building the evaluation capacity in our university by sustainable evaluation practices based on the evaluation guidelines. 
